# CoRSeq_V3-C_: a novel HIV-1 subtype C specific V3 sequence based coreceptor usage prediction algorithm

**DOI:** 10.1186/1742-4690-10-24

**Published:** 2013-02-27

**Authors:** Kieran Cashin, Lachlan R Gray, Martin R Jakobsen, Jasminka Sterjovski, Melissa J Churchill, Paul R Gorry

**Affiliations:** 1 Center for Virology, Burnet Institute, 85 Commercial Rd, Melbourne 3004VIC, Australia; 2 Department of Microbiology and Immunology, University of Melbourne, Parkville, VIC, Australia; 3 Departments of Biochemistry and Molecular Biology, Monash University, Melbourne, VIC, Australia; 4 Departments of Microbiology, Monash University, Melbourne, VIC, Australia; 5 Departments of Medicine, Monash University, Melbourne, VIC, Australia; 6 Departments of Infectious Diseases, Monash University, Melbourne, VIC, Australia; 7 Present address: Department of Biomedicine, Aarhus University, Aarhus, Denmark

**Keywords:** HIV-1, subtype C, gp120, V3, CCR5, CXCR4, Coreceptor, Prediction algorithm

## Abstract

**Background:**

The majority of HIV-1 subjects worldwide are infected with HIV-1 subtype C (C-HIV). Although C-HIV predominates in developing regions of the world such as Southern Africa and Central Asia, C-HIV is also spreading rapidly in countries with more developed economies and health care systems, whose populations are more likely to have access to wider treatment options, including the CCR5 antagonist maraviroc (MVC). The ability to reliably determine C-HIV coreceptor usage is therefore becoming increasingly more important. *In silico* V3 sequence based coreceptor usage prediction algorithms are a relatively rapid and cost effective method for determining HIV-1 coreceptor specificity. In this study, we elucidated the V3 sequence determinants of C-HIV coreceptor usage, and used this knowledge to develop and validate a novel, user friendly, and highly sensitive C-HIV specific coreceptor usage prediction algorithm.

**Results:**

We characterized every phenotypically-verified C-HIV gp120 V3 sequence available in the Los Alamos HIV Database. Sequence analyses revealed that compared to R5 C-HIV V3 sequences, CXCR4-using C-HIV V3 sequences have significantly greater amino acid variability, increased net charge, increased amino acid length, increased frequency of insertions and substitutions within the GPGQ crown motif, and reduced frequency of glycosylation sites. Based on these findings, we developed a novel C-HIV specific coreceptor usage prediction algorithm (CoRSeq_V3-C_), which we show has superior sensitivity for determining CXCR4 usage by C-HIV strains compared to all other available algorithms and prediction rules, including Geno2pheno_[coreceptor]_ and WebPSSM_SINSI_-C, which has been designed specifically for C-HIV.

**Conclusions:**

CoRSeq_V3-C_ is now openly available for public use at http://www.burnet.edu.au/coreceptor. Our results show that CoRSeq_V3-C_ is the most sensitive V3 sequence based algorithm presently available for predicting CXCR4 usage of C-HIV strains, without compromising specificity. CoRSeq_V3-C_ may be potentially useful for assisting clinicians to decide the best treatment options for patients with C-HIV infection, and will be helpful for basic studies of C-HIV pathogenesis.

## Background

Human immunodeficiency virus type 1 (HIV-1) gains entry into immune cells by binding to CD4 and one of two coreceptors, CCR5 or CXCR4 [1-4]. HIV-1 phenotypes are defined by the ability of HIV-1 to use CCR5 (R5), CXCR4 (X4) or both coreceptors (R5X4) for entry [5]. Transmission and establishment of HIV-1 infections is typically associated with R5 viruses. However, in 40-50% of individuals infected with subtype B HIV-1 (B-HIV), R5X4 and/or X4 viruses (collectively referred to hereafter as CXCR4-using viruses) emerge during disease progression [6,7]. The emergence of CXCR4-using viruses is associated with rapid CD4+ T-cell decline and accelerated onset of AIDS [8]. In contrast, individuals infected with subtype C HIV-1 (C-HIV) frequently harbor R5 viruses throughout all stages of disease [9] (reviewed in [10,11]). However, recent studies are reporting increased incidence of CXCR4-using C-HIV strains emerging at late stages of infection (up to 52%) [12-15], which may be associated with exposure to antiretroviral therapies (ART). Importantly, C-HIV infections constitute the majority of HIV-1 infections worldwide and are responsible for more than 95% of HIV-1 infections in Southern Africa, Central Asia and parts of South-East Asia, which are regions of the world where the HIV-1 pandemic is at its worst [11]. Furthermore, C-HIV is now spreading rapidly in more developed nations such as Brazil and neighboring countries [16].

The CCR5 antagonist maraviroc (MVC) is presently used as an antiretroviral (ARV) for treatment of HIV-1 infected subjects who have no detectable CXCR4-using virus in their plasma [17-19]. Thus, the ability to phenotypically characterize coreceptor usage of circulating HIV-1 strains has become clinically important. Currently, the gold standard for determining viral coreceptor usage is a cell entry assay with infectious viruses pseudotyped with patient-derived envelopes [20]. However, this method is expensive, labor intensive and time consuming and therefore, impractical in resource constrained regions where C-HIV predominates. Consequently, sequence based *in silico* predictive algorithms capable of predicting viral coreceptor usage have been developed, principally for B-HIV variants, offering a rapid, simplistic and inexpensive alternative to cell based entry assays.

Studies have shown that the major determinants of HIV-1 CCR5 and/or CXCR4 usage lie within the third variable loop (V3) of the viral surface envelope glycoprotein, gp120 [21-25]. Current coreceptor usage prediction algorithms exploit these V3 sequence characteristics in order to predict coreceptor specificity. Studies assessing the accuracy of currently available coreceptor usage prediction algorithms have shown limited success in regards to correctly predicting C-HIV coreceptor usage [26-31]. These algorithms will need significant improvements in order for MVC and other future CCR5 antagonists to become accessible for the vast majority of subjects with C-HIV infection. In this study, we aimed to develop and validate a novel, user friendly and highly specific C-HIV specific coreceptor usage prediction algorithm.

## Results

### C-HIV V3 sequence characteristics associated with coreceptor usage

In this study we analyzed all the phenotypically characterized patient-derived C-HIV V3 sequences currently available in the Los Alamos HIV Database in order to identify potential V3 sequence characteristics capable of differentiating CXCR4-using from R5 HIV-C Envs. We selected one representative CXCR4-using and/or R5 V3 sequence per subject in order to avoid biasing the results by the resampling of highly related sequences. In total, we assembled 69 unique CXCR4-using and 473 unique R5 C-HIV V3 sequences.

**Figure 1:**
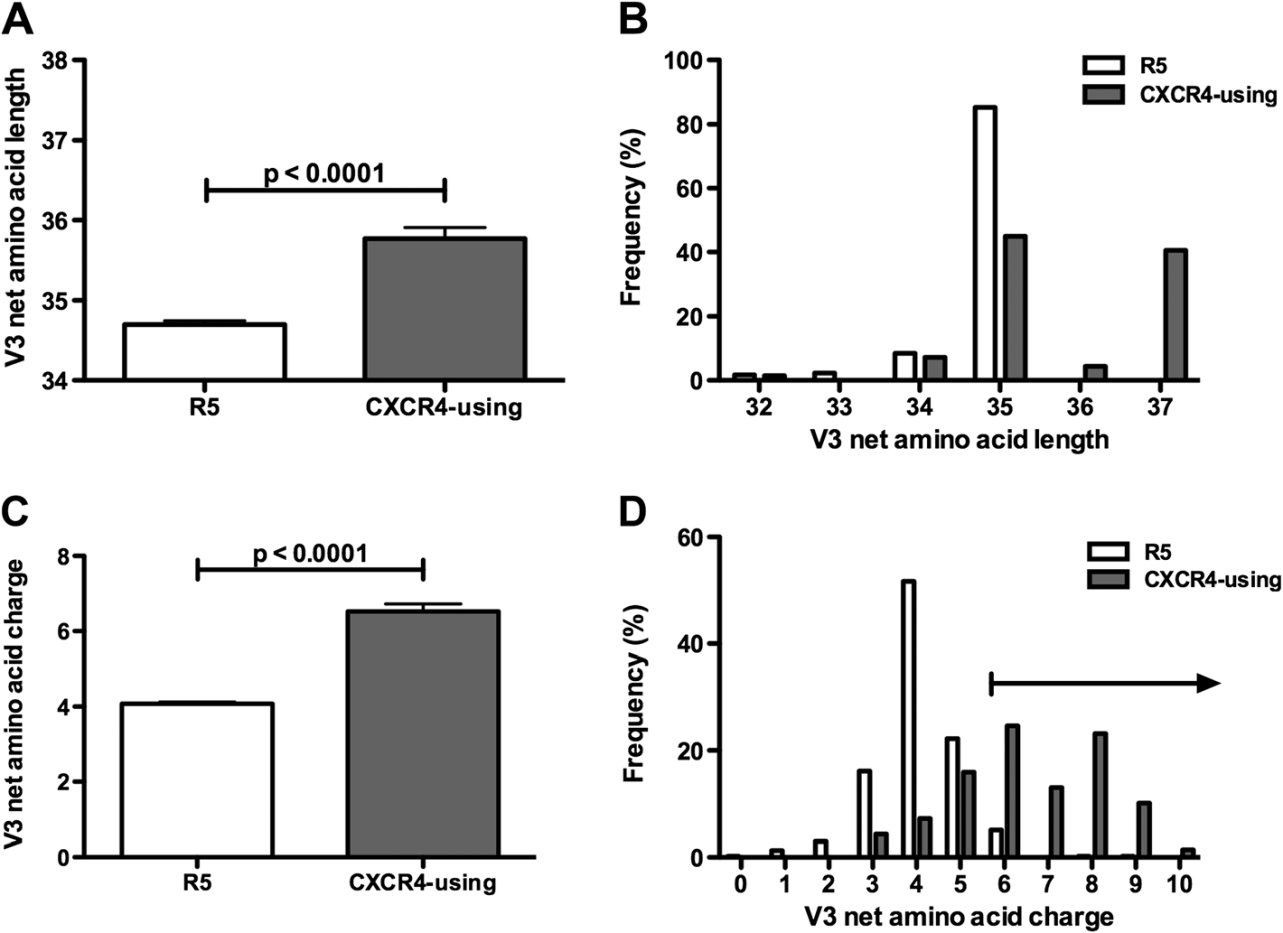
**V3 length and charge alterations associated with C-HIV coreceptor usage.** (**A**, **B**), A comparison of V3 amino acid lengths segregating CXCR4-using from R5 C-HIV strains. (**C**, **D**), A comparison of V3 charge alterations segregating CXCR4-using from R5 C-HIV strains.

CXCR4-using C-HIV V3 sequences were found to be significantly longer (range 32–37 amino acids) than the R5 C-HIV V3 sequences (range 32–35 amino acids; p<0.0001 by a Mann Whitney U test) (Figure 1A). Also, the net charge of CXCR4-using V3 sequences (range 3–10) was significantly higher than that of R5 V3 sequences (range 0–6; p<0.0001 by a Mann Whitney U test) (Figure 1C). Interestingly, the majority of CXCR4-using V3 sequences had a net charge of ≥6 (Figure 1D). The largely non-overlapping pattern of CXCR4-using and R5 V3 sequences suggests that a charge cutoff of ≥6 could be used to predict CXCR4-usage. Indeed, the V3 net charge of CXCR4-using C-HIV V3 sequences was significantly greater than that of the R5 C-HIV V3 sequences when a cutoff of ≥6 was considered (p<0.0001 by a Mann Whitney U test). In contrast, the significant overlap pattern between CXCR4-using and R5 V3 sequences with a net amino acid length of 35 (Figure 1B) suggests that V3 length may not be a characteristic sensitive enough to predict coreceptor usage of most C-HIV strains. However, a cutoff length of ≥36 amino acids could be used to predict some CXCR4-using C-HIV V3 sequences. Unlike previous studies, we found no association between coreceptor usage and V3 sequence hydrophobicity using the Kyte Doolittle hydrophobicity scale (data not shown) [14,29,32-35].

**Figure 2:**
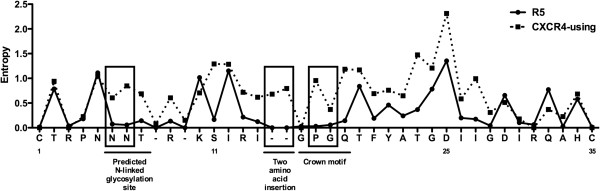
**Entropy plot comparing V3 sequence variability between CXCR4-using and R5 C-HIV Envs.** The potential N-linked glycosylation site, positions 1, 11, 25 and 35, the position of the two amino acid insertion, and the V3 GPGQ crown motif are highlighted. Boxes illustrate regions of specific interest, where there is variability within CXCR4-using V3 sequences but little or no variability within R5 V3 sequences.

We next produced an entropy plot to more precisely determine patterns of V3 variability between R5 and CXCR4-using C-HIV V3 sequences (Figure 2). Higher entropy values indicate greater variability at specific sites within V3. The CXCR4-using V3 sequences displayed greater overall variability than the R5 V3 sequences. Notably, the residues within the predicted N-glycosylation site (PNGS) at amino acid positions 6–8 were more conserved among R5 V3 sequences than CXCR4-using V3 sequences (Figure 2). Consistent with this observation, R5 V3 sequences had significantly more PNGS than CXCR4-using V3 sequences (p<0.0001 by a Mann Whitney U test); 20.3% of CXCR4-using V3 sequences lacked a PNGS at this motif, compared to only 2.5% of R5 V3 sequences (p=0.0002 by Fisher’s exact test). Residues 15, 16 and 17, located within the V3 crown motif, also showed greater variability in CXCR4-using V3 sequences compared with R5 V3 sequences (Figure 2). In fact, an alteration of at least one substitution within the V3 crown motif was significantly more frequent in CXCR4-using C-HIV V3 sequences (71%) than in R5 C-HIV V3 sequences (3.2%) (p<0.0001 by Fisher’s exact test). Furthermore, a two amino acid insertion immediately upstream of the V3 crown (Figure 2) was frequently present in CXCR4-using V3 sequences (42%), yet was never observed in R5 V3 sequences (p<0.0001 by Fisher’s exact test). Using mutagenesis studies, we recently showed that either a V3 crown substitution or a proximal two amino acid insertion were important for CXCR4-usage of C-HIV, individually conferring R5X4 phenotypes, and conferring an X4 phenotype when they were present together (M.R. Jakobsen, K. Cashin and P.R. Gorry, unpublished data). Notably, position 25 showed extensive variability in both R5 and CXCR4-using C-HIV V3 sequences (Figure 2).

Next we determined the type and frequency of amino acids at individual V3 positions, and compared the amino acid frequencies of CXCR4-using and R5 V3 sequences. Table 1 displays the amino acid alterations within V3 that either; (i) only occur in CXCR4-using or R5 V3 sequences, or (ii) exhibit variability that is significantly different between CXCR4-using and R5 V3 sequences, and thus may potentially be able to help discriminate CXCR4-using from R5 C-HIV viruses. We next assessed all of the amino acid alterations shown in Table 1 for their ability to increase the sensitivity of detecting CXCR4-using C-HIV viruses when tested in novel prototype coreceptor usage prediction algorithms. These analyses showed that the presence of Glu10, Ile11, Asn11, a gap at position 23, Thr25, a gap at position 25, or Glu32 or Phe34 (Table 1) could all individually contribute to increasing the sensitivity and/or specificity of detecting CXCR4-using C-HIV variants (Additional file 1). Interestingly, factoring in the presence or absence of a PNGS at amino acid positions 6–8 did not improve sensitivity or specificity (data not shown), despite being a significant parameter for distinguishing R5 and CXCR4-using sequences.

**Table 1:** V3 amino acid alterations associated with C-HIV coreceptor usage

**Mutation **^**a**^	**Phenotype**		**Fisher’s exact test (p value)**	**Mutation**	**Phenotype**		**Fisher’s exact test (p value)**
	**CCR5 usage**	**CXCR4 usage**			**CCR5 usage**	**CXCR4 usage**	
Asn6Tyr	0	4.3	ns	Gln18Arg	1.1	20.3	<0.0001
Asn7Lys	0	4.3	ns	Gln18His	0.4	13	0.0002
Thr8Ile	0.2	7.2	0.014	Gln18Lys	0	2.9	ns
Arg9Ile	0	5.8	0.0289	Phe20Val	0	7.2	0.0068
Lys10Glu	4.4	0	ns	Phe20Trp	0	2.9	ns
Ser11Arg	0.2	15.9	<0.0001	Thr23-	2.3	0	ns
Ser11Asn	0	4.3	ns	Thr23Arg	0	5.8	0.0289
Ser11His	0	2.9	ns	Gly24Lys	1.3	13	0.0013
Ser11Ile	0	2.9	ns	Asp25Arg	0.2	10.1	0.0015
Ser11Lys	0	1.4	ns	Asp25Lys	0.6	17.4	<0.0001
Ile12Lys	0	2.9	ns	Asp25Thr	0	7.2	0.0068
Arg13Asn	0	2.9	ns	Asp25His	0	1.4	ns
Arg13-	0.4	8.7	0.0067	Asp25-	2.5	4.3	ns
Ile14Leu	0.6	10.1	0.0015	Ile27Arg	0	7.2	0.0068
Ile14Thr	0	4.3	ns	Ile27Asn	0	2.9	ns
13-14 insertion	0	40.4	<0.0001	Gly28Lys	0	2.9	ns
Pro16Arg	0	37.7	<0.0001	Ile30Val	0	4.3	ns
Pro16Gln	0	4.3	ns	Gln32Glu	8	0	0.0068
Gly17Arg	0.4	8.7	0.0067	His34Phe	0	7.2	0.0068

^a^ Amino acid numbering is based on the consensus C-HIV V3 sequence.

Values are percentages of R5 or CXCR4-using C-HIV V3 sequences.

For these analyses, 69 unique CXCR4-using and 473 unique R5 C-HIV V3 sequences were included.

ns, not significant; -, deleted sequence.

### Other Env sequence characteristics may also distinguish CXCR4-using C-HIV strains from R5 C-HIV strains

We also performed a similar analysis of gp41 and all regions of gp120 outside of V3. Sequence characteristics that were significantly different between R5 and CXCR4-using C-HIV Envs included net amino acid charge in the V1 region (p=0.0306 by a Mann Whitney U test), V1 length (p=0.0415 by a Mann Whitney U Test), the number of PNGS within V1 (p=0.0363 by a Mann Whitney U Test), and length of the V4 region (p<0.0001 by a Mann Whitney U Test). These findings suggest that regions outside of the V3 loop may also be involved in determining coreceptor usage of C-HIV strains, and therefore could be used to predict coreceptor specificity. However, the limiting number of phenotypically characterised CXCR4-using C-HIV sequences containing regions other than V3 (n<40) may limit the significance of these findings, and also the practicality of including these characteristics in new C-HIV coreceptor usage prediction algorithms.

### Development of a novel C-HIV specific coreceptor usage prediction algorithm

**Figure 3:**
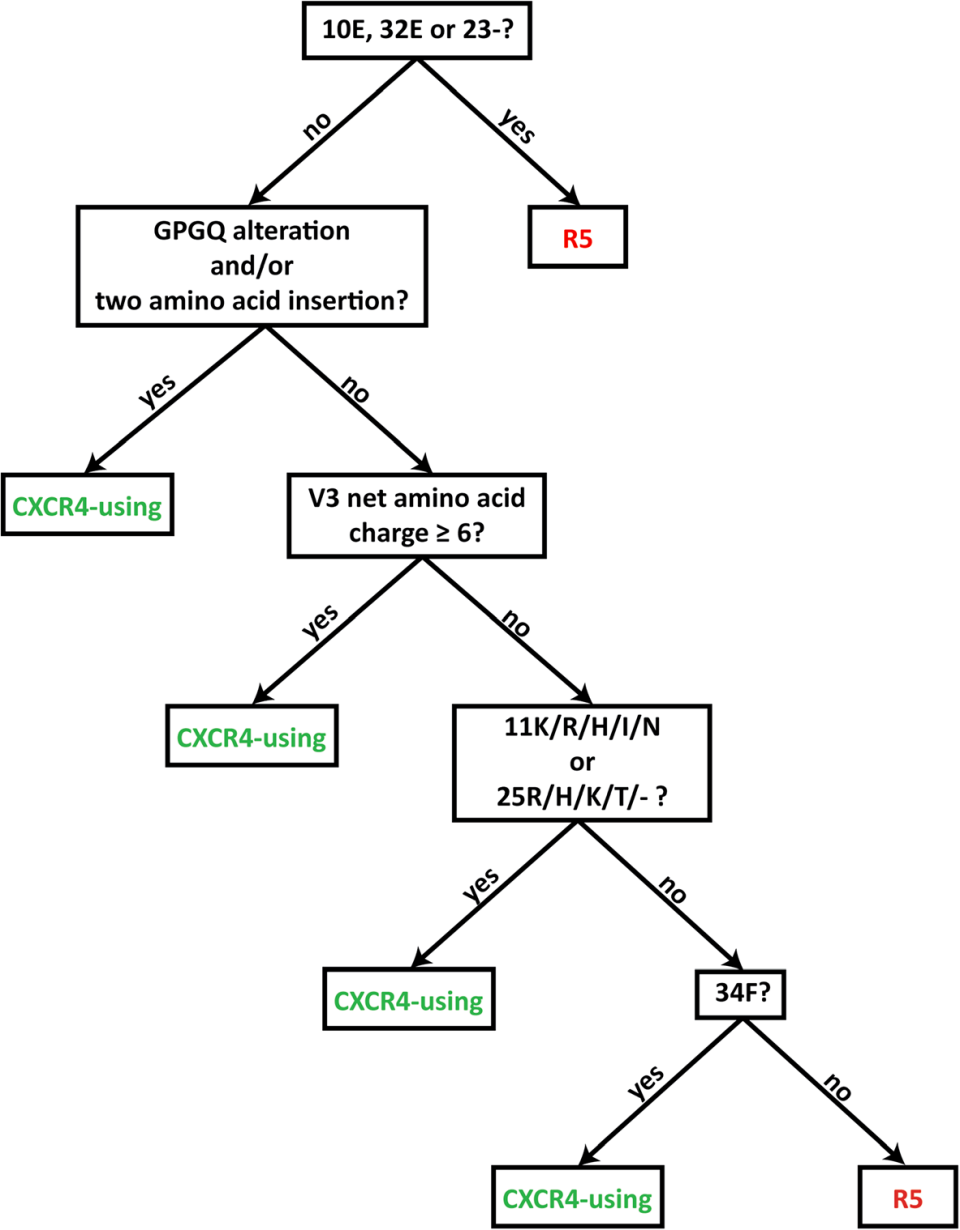
**A diagrammatic representation of the CoRSeq**_**V3-C **_**algorithm.** Query V3 sequences are aligned to the HXB2 V3 sequence and subjected to a sequential series of questions, which are ordered from top to bottom as indicated by arrows, and as described in detail in the Results section.

Next we developed a C-HIV specific V3 sequence based coreceptor usage prediction algorithm, founded principally on the V3 characteristics that we showed in the preceding studies could help distinguish CXCR4-using from R5 C-HIV viruses (CoRSeq_V3-C_). When a query V3 sequence is submitted to CoRSeq_V3-C_, which is now hosted at http://htpp://www.burnet.edu.au/coreceptor and presently available for use as a research tool, the sequence is aligned against the HXB2 V3 sequence, and a series of questions are addressed (Figure 3). For the purpose of sequence orientation and amino acid number assignment, HXB2 is the most practical reference sequence because, unlike the C-HIV consensus sequence, it contains the same number of amino acids as many CXCR4-using C-HIV Envs. First, the query sequence is assessed for the presence of amino acid alterations that occur exclusively in R5 C-HIV V3 sequences (Table 1), namely Glu10, Glu23 or a gap at position 23. If one or more of these amino acid alterations are present, the query sequence is determined to be that of a R5 C-HIV Env. If not, the query sequence is assessed for the presence of a number of signature CXCR4-using C-HIV V3 sequence characteristics and amino acid alterations that either (i) occur exclusively in CXCR4-using C-HIV V3 sequences, (ii) occur significantly more frequently in CXCR4-using C-HIV V3 sequences than in R5 C-HIV V3 sequences (Table 1), or (iii) improve the sensitivity and/or specificity of the algorithm for detecting CXCR4-using C-HIV variants (Additional file 1). Specifically, these CXCR4-using C-HIV signature alterations include an amino acid substitution within the GPGQ V3 crown motif and/or an immediately proximal two amino acid insertion, a net V3 amino acid charge of ≥6, and the presence of Lys11, Arg11, His11, Ile11, Asn11, Arg25, His25, Lys25, Thr25, a gap at position 25 or Phe34. If any of these traits are present the query sequence is determined to be that of a CXCR4-using C-HIV Env.

### Performance of CoRSeq_V3-C_ compared to other V3-based coreceptor usage prediction algorithms and tools

We next assessed the accuracy of CoRSeq_V3-C_ for predicting C-HIV coreceptor usage compared to several other prediction rules and algorithms (Table 2). We used two C-HIV V3 sequence data sets. Data set 1 was restricted to one representative V3 sequence per subject (69 CXCR4-using and 473 R5 Envs). Because CoRSeq_V3-C_ was developed using the V3 sequences of data set 1, we would anticipate a high sensitivity and specificity for our algorithm. Thus, we included a second data set in the study (data set 2), which consisted of every phenotypically characterized C-HIV V3 sequence currently available, allowing multiple sequences per patient (143 CXCR4-using and 1213 R5 C-HIV Envs). The other coreceptor usage prediction algorithms assessed were the Geno2pheno_[coreceptor]_ algorithm [36], the C-HIV specific WebPSSM algorithm (WebPSSM_SINSI_-C), two different B-HIV specific WebPSSM algorithms (WebPSSM_x4r5_-B and WebPSSM_SINSI_-B) [24,37], the 11/25 rule [25,38], the 11/24/25 rule [22,25,39,40], the 11/25/V3 charge rule [27], and the Lin et al., rule [41]. These rules and algorithms are described in the methods.

**Table 2:** Comparison of sensitivities and specificities of alternative algorithms and tools for predicting C-HIV coreceptor usage

**Coreceptor specificity prediction technique**	**One C-HIV V3 sequence per subject**		**All C-HIV V3 sequences**	
	**(Data set 1; 69 CXCR4-using and 473 R5)**		**(Data set 2; 143 CXCR4-using and 1213 R5)**	
	**Sensitivity (%)**	**Specificity (%)**	**Sensitivity (%)**	**Specificity (%)**
CoRSeq_V3-C_	94.20	91.12	90.85	99.98
Geno2pheno_[coreceptor]_	88.41	94.71	87.32	99.96
WebPSSM_SINSI_-C	88.41	90.7	88.02	99.86
WebPSSM_X4R5-_B	78.26	95.76	69.72	99.94
WebPSSM_SINSI_-B	59.42	99.36	57.04	99.98
11/25	46.38	98.94	47.87	99.99
11/24/25	56.52	97.25	52.12	99.99
11/25/V3 charge	79.71	88.16	77.47	99.96
Lin et al. rule	84.06	96.83	79.58	99.96

We found the 11/25 rule to be the least sensitive at correctly predicting CXCR4 usage in both data sets, with sensitivities of 46.38% and 47.87% for data sets 1 and 2, respectively (Table 2). Notably, the 11/24/25 rule showed comparatively improved sensitivity, with 56.52% and 52.12% for data sets 1 and 2, respectively. The 11/25/V3 charge rule showed further improvement, with 79.71% and 77.47% sensitivity for data sets 1 and 2, respectively. The Lin et al. rule had 84.06% and 79.58% sensitivity for data sets 1 and 2, respectively.

Among the more sophisticated V3 algorithms, Geno2pheno_[coreceptor]_ and WebPSSM_SINSI_-C both had sensitivities of 88.41% for data set 1, and sensitivities of 87.32% and 88.02% for data set 2, respectively. Geno2pheno_[coreceptor]_ was 94.71% specific for data set 1 and 99.96% specific for data set 2. WebPSSM_SINSI_-C was 90.7% specific for data set 1 and 99.86% specific for data set 2. The B-HIV specific WebPSSM_x4r5_-B and WebPSSM_SINSI_-B algorithms were comparatively inaccurate for C-HIV sequences, showing 78.26% and 59.42% sensitivities for data set 1, and 69.72% and 57.04% sensitivities for data set 2, respectively. In comparison, CoRSeq_V3-C_ was 94.2% sensitive and 91.12% specific for data set 1, and 90.85% sensitive and 99.98% specific for data set 2. Interestingly, the same CXCR4-using V3 sequences from data set 1 (n=4) and data set 2 (n=5) were incorrectly scored as R5 by all of the algorithms and rules investigated in this study, further highlighting that regions outside the V3 loop can confer CXCR4 usage, albeit in a minority of sequences. Together, our results show that CoRSeq_V3-C_ has superior sensitivity for detection of C-HIV CXCR4 usage compared to the best performing alternative algorithms, with comparable specificity.

## Discussion

In this study we conducted an extensive and comprehensive analysis of CXCR4-using and R5 C-HIV Envs in order to develop a novel C-HIV specific coreceptor usage prediction algorithm that is highly sensitive at predicting C-HIV CXCR4-usage. To do this we assembled every phenotypically characterized C-HIV Env V3 sequence available on the Los Alamos HIV Database and elucidated the specific sequence characteristics of V3 that differentiate R5 from CXCR4-using Envs. We found that CXCR4-using C-HIV V3 sequences have a significantly greater net charge and length compared to R5 Envs, consistent with the results of previous studies [22,23,29,41,42]. Crystal structure analysis and mutagenesis studies have shown that the V3 loop interacts directly with the coreceptor N-terminus and ECL2 region [43-45]. The surface of CXCR4 is more negatively charged than CCR5. Thus, Envs with a more positively charged V3 may have an enhanced interaction with CXCR4. We also found that CXCR4-using Envs are significantly less likely to have a PNGS within V3 than R5 Envs. Previous studies have suggested that the V3 N-terminal glycan enhances the interaction of gp120 with CCR5 [46-48]. Conversely, loss of this glycan has been shown to enhance viral CXCR4 usage [47,49].

Analysis of the amino acid type and frequency within V3, comparing R5 and CXCR4-using C-HIV Envs, revealed that CXCR4-using Envs have greater V3 variability than R5 Envs, consistent with previous studies [23,42]. Interestingly, the majority of CXCR4-using C-HIV Envs had either an amino acid substitution within the V3 GPGQ crown motif and/or a two amino acid substitution immediately proximal to the crown motif. The association between CXCR4-usage and a V3 crown motif alteration has been discussed in previous studies [27,29,41,42]. The V3 crown forms a beta turn secondary structure at the tip of the V3 loop. Recently, we constructed three-dimensional homology models of a primary X4 C-HIV gp120 with a GRGQ V3 crown motif and/or an Ile314-Gly315 insertion (M.R. Jakobsen, K .Cashin and P.R. Gorry, unpublished data), and showed that these alterations may potentially cause significant conformational changes in V3, which may enhance V3 flexibility and thus the interaction with CXCR4. V3 crown alterations and two amino acid insertions immediately proximal to the V3 crown occur more frequently in C-HIV CXCR4-using Envs than in any other HIV-1 subtype. However, analysis of 151 phenotypically characterized CXCR4-using and 225 R5 B-HIV V3 sequences obtained from the Los Alamos HIV database (one sequence per subject) revealed that a greater proportion of CXCR4-using B-HIV Envs have a two amino acid insertion (10%) immediately proximal to the V3 crown than R5 B-HIV Envs (0%). Therefore, although this two amino acid insertion occurs significantly less frequently in B-HIV CXCR4-using Envs than C-HIV CXCR4-using Envs (p<0.0001 by Fisher’s exact test), this defining characteristic of CXCR4-usage may therefore not be unique to C-HIV Envs. The presence of a V3 crown alteration was not significantly different between R5 (28%) and CXCR4-using (30%) B-HIV Envs.

We identified sequence characteristics in regions outside of V3 that are associated with coreceptor specificity, specifically in the V1 and V4 loops. Previous studies have described associations between sequence alterations within the V1/V2 loops and C-HIV coreceptor usage [32,50]. However, we recently showed that a V1 swap from a primary R5 C-HIV Env into an X4 C-HIV Env which developed in the same subject did not affect coreceptor usage, despite distinct sequence alterations in V1 segregating these two Envs (M.R. Jakobsen, K. Cashin and P.R. Gorry, unpublished data). Previous studies have suggested that extensive V1/V2 sequence mutations need to occur prior to the V3 mutations that are critical for CXCR4-usage of C-HIV [50,51]. Thus, the V1 swap experiment may have shown no affect on coreceptor usage because the V3 mutations necessary for CXCR4-usage were already present. Due to the limited number of phenotypically characterized full-length CXCR4-using C-HIV sequences, we did not conduct an amino acid type and frequency analysis outside the V3 region. However, Dimonte et al. [29] have conducted such a study, using C-HIV sequences predicted to use CXCR4 by Geno2pheno_[coreceptor]_, WebPSSM_SINSI_-C and 11/25/V3 charge. Here, the investigators identified multiple amino acids within gp41 that could be used to differentiate R5 from CXCR4-using C-HIV Envs, suggesting their inclusion in a coreceptor specificity prediction algorithm could improve predictive accuracy. However, a study by Thielen et al. [52] suggests that the inclusion of gp41 mutations does not substantially improve predictive accuracy.

A study by Raymond et al. [27] found the 11/25/V3 charge rule to be more accurate than the 11/25 rule, Geno2pheno_[coreceptor]_ and WebPSSM_SINSI_-C algorithms for predicting coreceptor specificity. While we found the 11/25/V3 charge rule to be more accurate than the 11/25 rule and the 11/24/25 rule, it was less accurate than Geno2pheno_[coreceptor]_, WebPSSM_SINSI_-C, CoRSeq_V3-C_ and the HIV-B specific WebPSSM_x4r5_-B algorithms. A recent study, by Lin et al. [41], utilizing 209 sequences isolated from 16 C-HIV infected subjects, also identified the association between C-HIV CXCR4 usage and a V3 crown alteration and/or a two amino acid insertion proximal to the V3 crown. Exploiting these V3 characteristics, the investigators were able to correctly predict 100% of the X4 Envs, 76.9% of the R5X4 Envs and 100% of the R5 Envs studied [41]. In our analysis, we found the Lin et al. rule to be more accurate than the 11/25 rule, the 11/24/25 rule, the 11/25/V3 charge rule and the HIV-B specific WebPSSM algorithms at predicting HIV-C CXCR4 usage. However, the Lin et al. rule was less accurate than the Geno2pheno_[coreceptor]_, WebPSSM_SINSI_-C, or CoRSeq_V3-C_ algorithms.

We developed CoRSeq_V3-C_ by determining the combination of V3 characteristics and amino acids specific to R5 or CXCR4-using C-HIV Envs that most accurately predict the coreceptor usage of all the currently available phenotypically characterized C-HIV V3 sequences. Future performance evaluation with additional phenotypically characterized and independent V3 sequences is required to more completely evaluate the sensitivity and specificity of CoRSeq_V3-C,_ which will be conducted as more C-HIV V3 sequences are deposited into the Los Alamos HIV database. We showed that CoRSeq_V3-C_ was maximally sensitive and specific for predicting CXCR4 usage when data was restricted to just one representative sequence per patient, which is a desirable quality. Importantly, as more C-HIV V3 sequences become available, CoRSeq_V3-C_ can be reviewed for the potential inclusion of other V3 characteristics and/or amino acids in order to further maximize its sensitivity and specificity. Thus, unlike previously developed algorithms, CoRSeq_V3-C_ is adaptable. CXCR4-using C-HIV Envs were first reported in patients on ART, and studies have suggested that ART provides a selective environment that may drive the emergence of CXCR4-using variants [53]. Thus, as ART become more widely available in C-HIV affected areas, adaptability may be an important quality for a C-HIV specific coreceptor specificity prediction algorithm.

## Conclusions

In summary, this study has advanced the genotypic characterization of C-HIV coreceptor usage. We elucidated the specific V3 sequence alterations associated with CXCR4-usage of C-HIV, and utilized these findings to develop an improved C-HIV specific V3 based coreceptor usage prediction algorithm. By comparison to the alternative predictive rules and algorithms, using two data sets that together comprise every available C-HIV V3 sequence, we have determined that CoRSeq_V3-C_ is presently the most sensitive algorithm for predicting CXCR4 usage of C-HIV, with specificity that remains comparable to the best performing alternative algorithms. Not only does CoRSeq_V3-C_ have the potential to be a useful tool for assisting clinicians to decide the best treatment options for patients with C-HIV infection, this new algorithm builds capacity for future studies of C-HIV pathogenesis.

## Methods

### C-HIV V3 sequence data sets

For this study we combined all phenotypically characterized C-HIV V3 sequences currently available in the Los Alamos Database (as of December 2012; n=1056), which were derived from diverse geographical locations such as Africa, India, South America, Europe, Israel and China with sequences from a longitudinal C-HIV study that we recently conducted (n=300) (M.R. Jakobsen, K. Cashin and P.R. Gorry, unpublished data). V3 sequences from Envs that were determined to use CXCR4 in cell entry assays or cause syncytia in MT-2 cells were labeled “CXCR4-using” sequences (n=143). V3 sequences from Envs that were determined to use CCR5 only in cell entry assays or did not induce syncytia in MT-2 cells were labeled “R5” sequences (n=1213).

### Coreceptor usage prediction rules and algorithms

The *in silico* algorithms assessed in this study were Geno2pheno_[coreceptor]_ using a false-positive rate of 10% (http://coreceptor.bioinf.mpi-inf.mpg.de/), as well as the C-HIV and B-HIV specific WebPSSM algorithms (http://indra.mullins.microbiol.washington.edu/webpssm/). The 11/25 rule predicts CXCR4-usage based on the presence of Arg, Lys or His at position 11 and/or 25 within the V3 region of gp120 [25,38]. The 11/24/25 rule predicts CXCR4-usage based on the presence of Arg, Lys or His at position 11, 24 and/or 25 within the gp120 V3 sequence [22,25,39,40]. The 11/25/V3 charge rule [27] predicts CXCR4-usage based on three criteria; (i) Arg/Lys at position 11 and/or Lys at position 25, (ii) Arg at position 25 and a net charge of ≥5, or (iii) a net charge of ≥6. The Lin et al. rule [41] predicts CXCR4-usage based on the presence of an alteration within the V3 GPGQ crown motif and/or the presence of a two amino acid insertion immediately proximal to the V3 crown, at positions 13 and 14. V3 net charge was calculated by subtracting the number of negatively charge amino acids (Asp and Glu) from the number of positively charged amino acids (Lys, Arg and His). Sensitivities were calculated by dividing the total number of correctly predicted CXCR4-using sequences by the total number of phenotypically characterised CXCR4-using sequences, and multiplying this number by 100. Specificities were calculated by dividing the number of correctly predicted R5 sequences by the total number of phenotypically characterised R5 sequences, and multiplying this number by 100.

### Sequence analyses

Sequence alignments were developed using CLC Main Workbench version 6.5. Sequence variability at each amino acid position was determined using the Los Alamos Database Entropy-One tool (http://www.hiv.lanl.gov/content/sequence/ENTROPY/entropy.html). Potential N-linked glycosylation sites were predicted using the Los Alamos Database N-Glycosite tool (http://www.hiv.lanl.gov/content/sequence/GLYCOSITE/glycosite.html).

### Statistical analysis

P-values were calculated using either a Fisher’s Exact test or a Mann Whitney U test. Values <0.05 were considered significant. All statistical tests were performed using Prism version 5.0 (GraphPad Software Inc., San Diego, CA).

## Competing interests

PRG is a member of the ViiV Australia Scientific Advisory Board, and has received honoraria from ViiV and financial support for travel to conferences. The other authors declare that they have no competing interests.

## Authors’ contributions

KC performed the sequence analysis. KC and LRG designed the algorithm. MRJ supplied HIV-1 sequences and helped interpret the results. JS, MJC and PRG helped interpret the results. KC and PRG wrote the manuscript. All authors helped edit the manuscript and have read and approved the final version.

## Supplementary Material

Additional file 1 Identification of amino acid alterations capable of improving the sensitivity or specificity of a prototype algorithm that predicts C-HIV CXCR4 usage based on the 11/25 rule, the presence of a V3 crown alteration and/or a two amino acid insertion. Click here for file

## References

[B1] Deng H Liu R Ellmeier W Choe S Unutmaz D Burkhart M Di Marzio P Marmon S Sutton RE Hill CM Identification of a major co-receptor for primary isolates of HIV-1 Nature 1996 381 6584 661 666 10.1038/381661a0 8649511

[B2] Dragic T Litwin V Allaway GP Martin SR Huang Y Nagashima KA Cayanan C Maddon PJ Koup RA Moore JP HIV-1 entry into CD4+ cells is mediated by the chemokine receptor CC-CKR-5 Nature 1996 381 6584 667 673 10.1038/381667a0 8649512

[B3] Feng Y Broder CC Kennedy PE Berger EA HIV-1 entry cofactor: functional cDNA cloning of a seven-transmembrane, G protein-coupled receptor Science 1996 272 5263 872 877 10.1126/science.272.5263.872 8629022

[B4] Choe H Farzan M Sun Y Sullivan N Rollins B Ponath PD Wu L Mackay CR LaRosa G Newman W The beta-chemokine receptors CCR3 and CCR5 facilitate infection by primary HIV-1 isolates Cell 1996 85 7 1135 1148 10.1016/S0092-8674(00)81313-6 8674119

[B5] Berger EA Doms RW Fenyo EM Korber BT Littman DR Moore JP Sattentau QJ Schuitemaker H Sodroski J Weiss RA A new classification for HIV-1 Nature 1998 391 6664 240 10.1038/34571 9440686

[B6] Connor RI Sheridan KE Ceradini D Choe S Landau NR Change in coreceptor use correlates with disease progression in HIV-1–infected individuals J Exp Med 1997 185 4 621 628 10.1084/jem.185.4.621 9034141 PMC2196142

[B7] Groot F van Capel TM Schuitemaker J Berkhout B de Jong EC Differential susceptibility of naive, central memory and effector memory T cells to dendritic cell-mediated HIV-1 transmission Retrovirology 2006 3 52 10.1186/1742-4690-3-52 16916447 PMC1562438

[B8] Vergis EN Mellors JW Natural history of HIV-1 infection Infect Dis Clin North Am 2000 14 4 809 825 v-vi 10.1016/S0891-5520(05)70135-5 11144640

[B9] Melby T Despirito M Demasi R Heilek-Snyder G Greenberg ML Graham N HIV-1 coreceptor use in triple-class treatment-experienced patients: baseline prevalence, correlates, and relationship to enfuvirtide response J Infect Dis 2006 194 2 238 246 10.1086/504693 16779731

[B10] Mathers CD Loncar D Projections of global mortality and burden of disease from 2002 to 2030 PLoS Med 2006 3 11 e442 10.1371/journal.pmed.0030442 17132052 PMC1664601

[B11] Jakobsen MR Ellett A Churchill MJ Gorry PR Viral tropism, fitness and pathogenicity of HIV-1 subtype C Futur Virol 2010 5 219 231 10.2217/fvl.09.77

[B12] Michler K Connell BJ Venter WD Stevens WS Capovilla A Papathanasopoulos MA Genotypic characterization and comparison of full-length envelope glycoproteins from South African HIV type 1 subtype C primary isolates that utilize CCR5 and/or CXCR4 AIDS Res Hum Retroviruses 2008 24 5 743 751 10.1089/aid.2007.0304 18507530

[B13] Connell BJ Michler K Capovilla A Venter WD Stevens WS Papathanasopoulos MA Emergence of X4 usage among HIV-1 subtype C: evidence for an evolving epidemic in South Africa AIDS 2008 22 7 896 899 10.1097/QAD.0b013e3282f57f7a 18427209

[B14] Cilliers T Nhlapo J Coetzer M Orlovic D Ketas T Olson WC Moore JP Trkola A Morris L The CCR5 and CXCR4 coreceptors are both used by human immunodeficiency virus type 1 primary isolates from subtype C J Virol 2003 77 7 4449 4456 10.1128/JVI.77.7.4449-4456.2003 12634405 PMC150635

[B15] Kassaye S Johnston E McColgan B Kantor R Zijenah L Katzenstein D Envelope coreceptor tropism, drug resistance, and viral evolution among subtype C HIV-1-infected individuals receiving nonsuppressive antiretroviral therapy J Acquir Immune Defic Syndr 2009 50 1 9 18 10.1097/QAI.0b013e31818ffdff 19295330 PMC2818215

[B16] Graf T Pinto AR The increasing prevalence of HIV-1 subtype C in Southern Brazil and its dispersion through the continent Virology 2013 435 1 170 178 10.1016/j.virol.2012.08.048 22999094

[B17] Dorr P Westby M Dobbs S Griffin P Irvine B Macartney M Mori J Rickett G Smith-Burchnell C Napier C Maraviroc (UK-427,857), a potent, orally bioavailable, and selective small-molecule inhibitor of chemokine receptor CCR5 with broad-spectrum anti-human immunodeficiency virus type 1 activity Antimicrob Agents Chemother 2005 49 11 4721 4732 10.1128/AAC.49.11.4721-4732.2005 16251317 PMC1280117

[B18] Hunt JS Romanelli F Maraviroc, a CCR5 coreceptor antagonist that blocks entry of human immunodeficiency virus type 1 Pharmacotherapy 2009 29 3 295 304 10.1592/phco.29.3.295 19249948

[B19] Gorry PR Ellett A Lewin SR Grayson L, Crowe S, McCarthy J, Mills J, Mouton J, Norrby SR, Paterson D, Pfaller M Maraviroc Kucers’ The Use of Antibiotics 2010 6 London: Hodder & Stoughton Ltd 2869 2876

[B20] Wilkin TJ Goetz MB Leduc R Skowron G Su Z Chan ES Heera J Chapman D Spritzler J Reeves JD Reanalysis of coreceptor tropism in HIV-1-infected adults using a phenotypic assay with enhanced sensitivity Clin Infect Dis 2011 52 7 925 928 10.1093/cid/cir072 21427401 PMC3106234

[B21] Hwang SS Boyle TJ Lyerly HK Cullen BR Identification of the envelope V3 loop as the primary determinant of cell tropism in HIV-1 Science 1991 253 5015 71 74 10.1126/science.1905842 1905842

[B22] De Jong JJ De Ronde A Keulen W Tersmette M Goudsmit J Minimal requirements for the human immunodeficiency virus type 1 V3 domain to support the syncytium-inducing phenotype: analysis by single amino acid substitution J Virol 1992 66 11 6777 6780 1404617 10.1128/jvi.66.11.6777-6780.1992 PMC240176

[B23] Hoffman NG Seillier-Moiseiwitsch F Ahn J Walker JM Swanstrom R Variability in the human immunodeficiency virus type 1 gp120 Env protein linked to phenotype-associated changes in the V3 loop J Virol 2002 76 8 3852 3864 10.1128/JVI.76.8.3852-3864.2002 11907225 PMC136063

[B24] Jensen MA Li FS van ’t Wout AB Nickle DC Shriner D He HX McLaughlin S Shankarappa R Margolick JB Mullins JI Improved coreceptor usage prediction and genotypic monitoring of R5-to-X4 transition by motif analysis of human immunodeficiency virus type 1 env V3 loop sequences J Virol 2003 77 24 13376 13388 10.1128/JVI.77.24.13376-13388.2003 14645592 PMC296044

[B25] Fouchier RA Groenink M Kootstra NA Tersmette M Huisman HG Miedema F Schuitemaker H Phenotype-associated sequence variation in the third variable domain of the human immunodeficiency virus type 1 gp120 molecule J Virol 1992 66 5 3183 3187 1560543 10.1128/jvi.66.5.3183-3187.1992 PMC241084

[B26] Recordon-Pinson P Soulie C Flandre P Descamps D Lazrek M Charpentier C Montes B Trabaud MA Cottalorda J Schneider V Evaluation of the genotypic prediction of HIV-1 coreceptor use versus a phenotypic assay and correlation with the virological response to maraviroc: the ANRS GenoTropism study Antimicrob Agents Chemother 2010 54 8 3335 3340 10.1128/AAC.00148-10 20530226 PMC2916345

[B27] Raymond S Delobel P Mavigner M Ferradini L Cazabat M Souyris C Sandres-Saune K Pasquier C Marchou B Massip P Prediction of HIV type 1 subtype C tropism by genotypic algorithms built from subtype B viruses J Acquir Immune Defic Syndr 2010 53 2 167 175 10.1097/QAI.0b013e3181c8413b 19996764

[B28] Garrido C Roulet V Chueca N Poveda E Aguilera A Skrabal K Zahonero N Carlos S Garcia F Faudon JL Evaluation of eight different bioinformatics tools to predict viral tropism in different human immunodeficiency virus type 1 subtypes J Clin Microbiol 2008 46 3 887 891 10.1128/JCM.01611-07 18199789 PMC2268339

[B29] Dimonte S Babakir-Mina M Mercurio F Di Pinto D Ceccherini-Silberstein F Svicher V Perno CF Selected amino acid changes in HIV-1 subtype-C gp41 are associated with specific gp120(V3) signatures in the regulation of co-receptor usage Virus Res 2012 168 1–2 73 83 22732432 10.1016/j.virusres.2012.06.019

[B30] Raymond S Delobel P Mavigner M Cazabat M Souyris C Sandres-Saune K Cuzin L Marchou B Massip P Izopet J Correlation between genotypic predictions based on V3 sequences and phenotypic determination of HIV-1 tropism AIDS 2008 22 14 F11 F16 10.1097/QAD.0b013e32830ebcd4 18753930

[B31] Delgado E Fernandez-Garcia A Vega Y Cuevas T Pinilla M Garcia V Sanchez M Gonzalez M Sanchez AM Thomson MM Evaluation of genotypic tropism prediction tests compared with in vitro co-receptor usage in HIV-1 primary isolates of diverse subtypes J Antimicrob Chemother 2012 67 1 25 31 10.1093/jac/dkr438 22010208

[B32] Singh A Page T Moore PL Allgaier RL Hiramen K Coovadia HM Walker BD Morris L Ndung’u T Functional and genetic analysis of coreceptor usage by dualtropic HIV-1 subtype C isolates Virology 2009 393 1 56 67 10.1016/j.virol.2009.07.021 19695656 PMC3492694

[B33] Choge I Cilliers T Walker P Taylor N Phoswa M Meyers T Viljoen J Violari A Gray G Moore PL Genotypic and phenotypic characterization of viral isolates from HIV-1 subtype C-infected children with slow and rapid disease progression AIDS Res Hum Retroviruses 2006 22 5 458 465 10.1089/aid.2006.22.458 16706624

[B34] McCormack GP Glynn JR Crampin AC Sibande F Mulawa D Bliss L Broadbent P Abarca K Ponnighaus JM Fine PE Early evolution of the human immunodeficiency virus type 1 subtype C epidemic in rural Malawi J Virol 2002 76 24 12890 12899 10.1128/JVI.76.24.12890-12899.2002 12438614 PMC136717

[B35] Ndung’u T Sepako E McLane MF Chand F Bedi K Gaseitsiwe S Doualla-Bell F Peter T Thior I Moyo SM HIV-1 subtype C in vitro growth and coreceptor utilization Virology 2006 347 2 247 260 10.1016/j.virol.2005.11.047 16406460

[B36] Lengauer T Sander O Sierra S Thielen A Kaiser R Bioinformatics prediction of HIV coreceptor usage Nat Biotechnol 2007 25 12 1407 1410 10.1038/nbt1371 18066037

[B37] Jensen MA Coetzer M van ’t Wout AB Morris L Mullins JI A reliable phenotype predictor for human immunodeficiency virus type 1 subtype C based on envelope V3 sequences J Virol 2006 80 10 4698 4704 10.1128/JVI.80.10.4698-4704.2006 16641263 PMC1472078

[B38] Shioda T Levy JA Cheng-Mayer C Small amino acid changes in the V3 hypervariable region of gp120 can affect the T-cell-line and macrophage tropism of human immunodeficiency virus type 1 Proc Natl Acad Sci U S A 1992 89 20 9434 9438 10.1073/pnas.89.20.9434 1409653 PMC50146

[B39] Cardozo T Kimura T Philpott S Weiser B Burger H Zolla-Pazner S Structural basis for coreceptor selectivity by the HIV type 1 V3 loop AIDS Res Hum Retroviruses 2007 23 3 415 426 10.1089/aid.2006.0130 17411375

[B40] Rosen O Sharon M Quadt-Akabayov SR Anglister J Molecular switch for alternative conformations of the HIV-1 V3 region: implications for phenotype conversion Proc Natl Acad Sci U S A 2006 103 38 13950 13955 10.1073/pnas.0606312103 16966601 PMC1599894

[B41] Lin NH Becerril C Giguel F Novitsky V Moyo S Makhema J Essex M Lockman S Kuritzkes DR Sagar M Env sequence determinants in CXCR4-using human immunodeficiency virus type-1 subtype C Virology 2012 433 2 296 307 10.1016/j.virol.2012.08.013 22954962 PMC3616623

[B42] Coetzer M Cilliers T Ping LH Swanstrom R Morris L Genetic characteristics of the V3 region associated with CXCR4 usage in HIV-1 subtype C isolates Virology 2006 356 1–2 95 105 16942785 10.1016/j.virol.2006.07.030

[B43] Brelot A Heveker N Adema K Hosie MJ Willett B Alizon M Effect of mutations in the second extracellular loop of CXCR4 on its utilization by human and feline immunodeficiency viruses J Virol 1999 73 4 2576 2586 10074102 10.1128/jvi.73.4.2576-2586.1999 PMC104012

[B44] Doranz BJ Lu ZH Rucker J Zhang TY Sharron M Cen YH Wang ZX Guo HH Du JG Accavitti MA Two distinct CCR5 domains can mediate coreceptor usage by human immunodeficiency virus type 1 J Virol 1997 71 9 6305 6314 9261347 10.1128/jvi.71.9.6305-6314.1997 PMC191903

[B45] Huang CC Tang M Zhang MY Majeed S Montabana E Stanfield RL Dimitrov DS Korber B Sodroski J Wilson IA Structure of a V3-containing HIV-1 gp120 core Science 2005 310 5750 1025 1028 10.1126/science.1118398 16284180 PMC2408531

[B46] Pollakis G Abebe A Kliphuis A Chalaby MI Bakker M Mengistu Y Brouwer M Goudsmit J Schuitemaker H Paxton WA Phenotypic and genotypic comparisons of CCR5- and CXCR4-tropic human immunodeficiency virus type 1 biological clones isolated from subtype C-infected individuals J Virol 2004 78 6 2841 2852 10.1128/JVI.78.6.2841-2852.2004 14990703 PMC353763

[B47] Polzer S Dittmar MT Schmitz H Schreiber M The N-linked glycan g15 within the V3 loop of the HIV-1 external glycoprotein gp120 affects coreceptor usage, cellular tropism, and neutralization Virology 2002 304 1 70 80 10.1006/viro.2002.1760 12490404

[B48] Clevestig P Pramanik L Leitner T Ehrnst A CCR5 use by human immunodeficiency virus type 1 is associated closely with the gp120 V3 loop N-linked glycosylation site J Gen Virol 2006 87 Pt 3 607 612 16476981 10.1099/vir.0.81510-0

[B49] Nabatov AA Pollakis G Linnemann T Kliphius A Chalaby MI Paxton WA Intrapatient alterations in the human immunodeficiency virus type 1 gp120 V1V2 and V3 regions differentially modulate coreceptor usage, virus inhibition by CC/CXC chemokines, soluble CD4, and the b12 and 2G12 monoclonal antibodies J Virol 2004 78 1 524 530 10.1128/JVI.78.1.524-530.2004 14671134 PMC303404

[B50] Coetzer M Nedellec R Cilliers T Meyers T Morris L Mosier DE Extreme genetic divergence is required for coreceptor switching in HIV-1 subtype C J Acquir Immune Defic Syndr 2011 56 1 9 15 10.1097/QAI.0b013e3181f63906 20921899 PMC3006070

[B51] Pastore C Nedellec R Ramos A Pontow S Ratner L Mosier DE Human immunodeficiency virus type 1 coreceptor switching: V1/V2 gain-of-fitness mutations compensate for V3 loss-of-fitness mutations J Virol 2006 80 2 750 758 10.1128/JVI.80.2.750-758.2006 16378977 PMC1346864

[B52] Thielen A Lengauer T Swenson LC Dong WW McGovern RA Lewis M James I Heera J Valdez H Harrigan PR Mutations in gp41 are correlated with coreceptor tropism but do not improve prediction methods substantially Antivir Ther 2011 16 3 319 328 10.3851/IMP1769 21555814

[B53] Delobel P Sandres-Saune K Cazabat M Pasquier C Marchou B Massip P Izopet J R5 to X4 switch of the predominant HIV-1 population in cellular reservoirs during effective highly active antiretroviral therapy J Acquir Immune Defic Syndr 2005 38 4 382 392 10.1097/01.qai.0000152835.17747.47 15764954

