# Supplementary material for: CoRSeqV3-C: a novel HIV-1 subtype C specific V3 sequence based coreceptor usage prediction algorithm
Source: Retrovirology. 2013 Feb 27;10:24. doi: 10.1186/1742-4690-10-24 (PMC3599735; doi:10.1186/1742-4690-10-24)
Supplement: Additional file 1 — Identification of amino acid alterations capable of improving the sensitivity or specificity of a prototype algorithm that predicts C-HIV CXCR4 usage based on the 11/25 rule, the presence of a V3 crown alteration and/or a two amino acid insertion. [file 1742-4690-10-24-S1.pdf]

CoRSeq$_{V3-C}$: A novel HIV-1 subtype C specific V3 sequence based coreceptor usage prediction algorithm.

Kieran Cashin, Lachlan R. Gray, Martin R. Jakobsen, Jasminka Sterjovski, Melissa J. Churchill and Paul R. Gorry.

Supplementary Table 1.

Identification of amino acid alterations capable of improving the sensitivity or specificity of a prototype algorithm that predicts C-HIV CXCR4 usage based on the 11/25 rule, the presence of a V3 crown alteration and/or a two amino acid insertion.

Algorithm	Sensitivity (%)	Specificity (%)
**11/25 rule, crown alteration and/or two amino acid insertion only**	**84.1**	**96.83**
+ Glu10	84.1	97
+ Ile11	85.5	96.83
+ Asn11	85.5	96.83
+ Gap23	84.1	97
+ Thr25	87	96.83
+ Gap25	85.5	94.5
+ Glu32	84.1	97
+ Phe34	85.5	96.83
